# Supplementary material for: Real-World Experience of Olaparib Maintenance in High-Grade Serous Recurrent Ovarian Cancer Patients with BRCA1/2 Mutation: A Korean Multicenter Study
Source: J Clin Med. 2019 Nov 8;8(11):1920. doi: 10.3390/jcm8111920 (PMC6912318; doi:10.3390/jcm8111920)
Supplement: Supplementary file 1 [file jcm-08-01920-s001.zip › table S1.docx]

**Table S1.** Long-term responders on olaparib maintenance.

| **No.** | **Olaprib maintenance period, months** | **Age, years** | **Initial FIGO stage** | **Initial residual status** | ***BRCA* mutation status** | **Platinum-free interval,**  **months** | **Objective response to most recent chemotherapy** | **Previous chemotherapy regimen** | **Best objective response with olaparib** | **Adverse event and management** |
| --- | --- | --- | --- | --- | --- | --- | --- | --- | --- | --- |
| 1 | 30.2 | 40 | IV | >1cm | *BRCA1* | 14.6 | Complete | 2 (Taxane/carboplatin – Pegylated liposomal doxorubicin /carboplatin) |  | Nausea/vomiting – 50% DR |
| 2 | 34.8 | 49 | IIIC | 0.1~1cm | *BRCA2* | 36.0 | Partial | 2 (Taxane/carboplatin – Taxane/carboplatin) | CR | Nausea/vomiting – 50% DR |
| 3 | 35.4 | 53 | IIIC | 0.1~1cm | *BRCA2* | 11.7 | Partial | 2 (Taxane/carboplatin – Pegylated liposomal doxorubicin /carboplatin) | CR |  |
| 4 | 35.7 | 62 | IIIB | No residual | *BRCA1* | 9.5 | partial | 2 (Taxane/carboplatin – Pegylated liposomal doxorubicin /carboplatin) | CR |  |
|  |  |  |  |  |  |  |  |  |  |  |

CR: complete remission, DR : dose reduction
