# Supplementary material for: Real-World Experience of Olaparib Maintenance in High-Grade Serous Recurrent Ovarian Cancer Patients with BRCA1/2 Mutation: A Korean Multicenter Study
Source: J Clin Med. 2019 Nov 8;8(11):1920. doi: 10.3390/jcm8111920 (PMC6912318; doi:10.3390/jcm8111920)
Supplement: Supplementary file 1 [file jcm-08-01920-s001.zip › table S2.docx]

**Table S2.** Characteristics of patients with recurrence after olaparib maintenance therapy (*N*=37).

| **Characteristics** | ***N*=37** |
| --- | --- |
| **Age, years** |  |
| Median (Range) | 53 (29~70) |
|  |  |
| **Initial FIGO stage, n(%)** |  |
| IA | 1 (2.7) |
| IC | 1 (2.7) |
| IIIA | 2 (5.4) |
| IIIB | 1 (2.7) |
| IIIC | 16 (43.2) |
| IV | 16 (43.2) |
|  |  |
| **Initial residual status, n(%)** |  |
| No residual | 14 (37.8) |
| 0.1~1cm | 19 (51.4) |
| >1cm | 4 (10.8) |
|  |  |
|  |  |
| **Platinum-free interval,**  **Duration, n (%)** |  |
| 6-12 months | 14 (37.8) |
| >12 months | 23 (62.2) |
| **Median (range), months** | 12.9 (6.0~48.0) |
|  |  |
|  |  |
| **Objective response to most recent chemotherapy, n (%)** |  |
| Complete | 19 (51.4) |
| Partial | 18 (48.6) |
|  |  |
| **Number of previous chemotherapy regimen, no (%)** |  |
| 2 | 21 (56.8) |
| 3 | 11 (29.7) |
| 4 | 3 (8.1) |
| ≥5 | 2 (5.4) |
| **Median (range)** | 2 (2~13) |
|  |  |
| ***BRCA* mutation status, no (%)** |  |
| **Germline** |  |
| *BRCA1* | 30 (81.1) |
| *BRCA2* | 4 (10.8) |
| *BRCA1/2* | 1 (2.7) |
| **Somatic** |  |
| *BRCA1/2* | 2 (5.4) |
|  |  |

FIGO : International Federation of Gynecology and Obstetrics
