# Supplementary figures and images for: Real-World Experience of Olaparib Maintenance in High-Grade Serous Recurrent Ovarian Cancer Patients with BRCA1/2 Mutation: A Korean Multicenter Study
Source: J Clin Med. 2019 Nov 8;8(11):1920. doi: 10.3390/jcm8111920 (PMC6912318; doi:10.3390/jcm8111920)

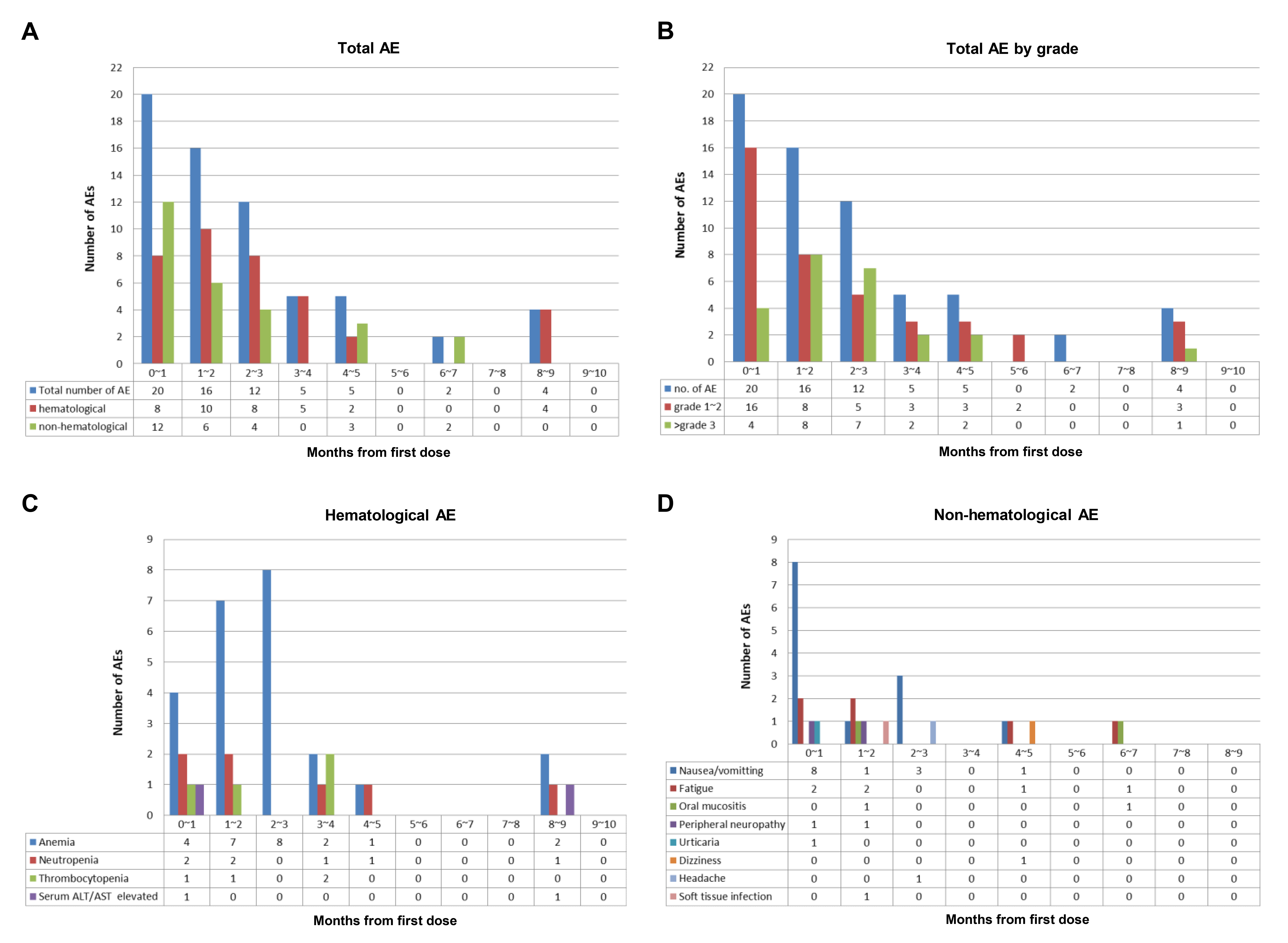

Supplement: Supplementary file 1 [file jcm-08-01920-s001.zip › figure S1.tif]
